# A System of Practical Medicine

**Published:** 1885-03

**Authors:** 


A System of Practical Medicine, by American Authors. Edited by William Pepper, M. D., LL.D., Provost and Professor of the Theory and Practice of Medicine and of Clinical Medicine in the University of Penn.; assisted by Louis Starr, M. D., Clinical Professor of Diseases of Children in the hospital of the University of Pennsylvania. Volume 1, pp. 1094. Pathology and general diseases. Philadelphia: Lea Brothers & Co. 1885.

The general arrangement of the subjects in this work is upon the principle adopted of late in some of the comprehensive treatises on surgery. Distinct branches are treated by individuals who are recognized as having made special investigation of the respective topics; and thus a strong presumption is raised in favor of the competency of the several authors of the contributions. Most of the writers in the present volume are well and favorably known to the medical public, and their prestige affords a guarantee for the most thorough elaboration of the various subjects.

The standpoint from which each contributor views his separate department, must of necessity give a coloring to all its surroundings, and impart more or less of the characteristics which pertain to a specialty. That exclusiveness and dogmatism which is often associated with attention to the details of a single inquiry, are precluded in the present undertaking by the selection of those whose range of thought is not limited to their prescribed fields of investigation; and there is not a writer upon any subject thus far, who should be considered as a specialist in the matter presented. This fact does not imply a want of familiarity with all the bearings of the subject, and affords a vantage-ground from which to compare his observations and opinions with those of others who have been occupied in the same sphere of investigation.

That exceptional statements may occur in the course of any individual disquisition upon a given subject does not show a want of judgment on the part of the author, nor does it indicate incapacity to grapple with the principles involved, as the outlook is so varied by surrounding circumstances that even statistics lead to conclusions which cannot be accepted as truths.

Upon examination of the very valuable contents of this volume the reader must be prepared therefore, to encounter some things which are not entirely as they should be; but at the same time it must be allowed that its trustworthiness in all essentials entitles the work to our confidence. If we accept the title of "a system of practical medicine" as appropriate for this undertaking, a difficulty arises in designating the useful and otherwise instructive data included in the first 227 pages under this heading.

Should the caption succeeding this portion of the book, as "General Diseases, from special morbid agents operating from without," be construed to refer all of this list to bacterial origin, it should have been more definitely set forth than is done in the preface.

The subjects treated are as follows: General Morbid Processes, by Reginald H. Fitz, M. D.; General Etiology, Medical Diagnosis and Prognosis, by Henry Hartshorn, M. D.; Hygiene, by John S. Billings, A. M., M. D., LL. D.; Drainage and Sewage in their Hygienic Relations, by George E. Waring, Jr. M. Inst., C. E.; Simple Continued Fever, by J. H. Hutchinson, M. D.; Typhoid Fever, by J. H. Hutchinson, M. D.; Typhus Fever, by J. H. Hutchinson, M. D.; Relapsing Fever, by William Pepper, M. D.; Variola, by James Nevins Hyde, M. D.; Vaccinia, by Frank P. Foster, M. D.; Varicella, by James Nevins Hyde, M. D.; Scarlet Fever, by J. Lewis Smith, M. D.; Rubeola, by W. A. Hardaway, A.

M., M. D.; Rotheln, by W. A. Hardaway, A. M., M. D.; Malarial Fever, by Samuel Bemiss, M. D.; Parotitis, by John M. Keating, M. D.; Erysipelas, by James Nevins Hyde, M. D.; Yellow Fever, by Samuel M. Bemiss, M. D.; Diphtheria, by Abraham Jacobi, M. D.; Cholera, by Alfred Stillè, M. D., LL. D; Plague, by James C. Wilson, A. M., M. D.; Leprosy, by James C. White, M. D; Epidemic Cerebro-spinal Meningitis, by A. Stillè, M. D., LL. D.; Pertussis, by John M. Keating, M. D.; Influenza, by James C. Wilson, A. M., M. D.; Dengue, by H. D. Schmidt, M. D.; Rabies and Hydrophobia, by James Law, F. R., C. V. S.; Glanders and Farcy, by James Law, F. R., C. V. S.; Anthrax (Malignant Pustule,) by James Law, F. R., C. V. S.; Pyæmia and Septicæmia, by B. A. Watson, A. M., M. D.; Puerperal Fever, by William T. Lusk, M. D.; Beriberi, by Duane B. Simmons, M. D.

While the readers of this book must feel some disappointment in not finding other views than those of a scientific engineer upon "Drainage and Sewerage in their Hygienic Relations," this lack of medical judgment is remedied by the authorized and pointed *ex cathedra* delivery of the author on hygiene, which might have succeeded, instead of preceding, this business paper. With the recognized capacity of the editor in the clinical department, it was to have been expected that his contributions should not have been confined to a single article in the practical department; but his modesty in this respect has yielded good results in the selection of an able corps of collaborators in the various departments, and in leaving the responsibility with each writer as to the great principles of causation and development of diseased action in the organism.

In view of the transition state of medical pathology, and the difference of opinions among authorities, as to morbid processes, much remains *sub judice.* The bacillus and spirillum play a prominent part in many of the contributions; yet it doth not yet appear that the causative element which is operative in all these diseases can be traced to outside influences, even excluding spores, micrococci, or other germs of the bacterial family.

It is evident that a proper appreciation of the several contributions cannot be given in the limited space which can be devoted to this review. Even full abstracts could not do justice to the concise presentation of some of the topics; and hence we must limit our notice to a few of the more salient points which strike us as worthy of comment.

The adherence to the term typhoid fever is based more upon the fact of its general use than upon any recognition of its fitness in expressing the characteristics of this enteric fever; and, though the term used by Wood may be based upon the effects rather than the constituent elements of disease, the real causative influence of typhoid fever is involved in so much doubt that such an invariable accompaniment of its progress as the intestinal phenomena might well serve to characterize this disease. The attempt of this writer, as of others, to trace typhoid fever to fœcal emanations or sewer gas, is not sustained by the observations of many practitioners, who fail to detect such an agency in numerous cases that occur under circumstances free from all such contamination. While spirits of turpentine is allowed a prominent place in the means of cure, it is clear that it is not given the place of preëminent utility to which it is entitled, and perhaps the great majority of cases of this form of disease may be treated more successfully by it, in combination with carbonate of ammonia, camphor and opium, in a mucilaginous mixture, than by any and all other means at our command. In that adynamic hemorrhage from the bowels, for which our author suggests, among other astringents, the acetate of lead, it has been found especially useful in conjunction with opium and the sulphate of quinine, giving a pill compounded of sulph. quin., 2 grs., ac.

plumb. 1 gr., and opium ½ gr. every two hours until the hemorrhagic feature is controlled. In this, as in other therapeutic combinations, the chemical relations may be disregarded.

For the relief or prevention of the bed sores, nothing equals the application of collodium f℥i, tinct. of iodine fʒi and camphor ʒi, with a camel's hair pencil passed over the irritated surface daily, so as to give it a coating.

The almost hopeless aspect of relapsing fever, presented by the editor, with opiates as the sole redeeming feature, in the treatment of symptoms, leaves the way open for a simple remedy which has been given with a favorable result:

| | |
|---|---|
| ℞. Infusion of Gentian, . . . . . . . . . . . . . | f ℥ x. |
| Aqueous Tinct. of Rhubarb,. . . . . . . . . . . . | f ℥ ii. |
| Tinct. of Nux Vomica, . . . . . . . . . . . . . . | f ʒ ss. |
| Bicarb. of Soda, . . . . . . . . . . . . . . . . | ʒ i. |

Mix and give half a wineglass every two or three hours.

The recognized efficacy of the muriated tincture of iron in erysipelas is urged in connection with other measures of treatment by the contributor of this paper; yet he makes an allusion to the hypodermic injection of the carbolic acid as wanting in efficacy, which has been resorted to by others with the most satisfactory results locally and constitutionally.

In the article on yellow fever no reference is made to the practice of Dr. R. H. Day, published some years ago in the New Orleans *Medical Journal*, claiming that the disease was jugulated by combining 10 grains of sulph. quinine with 5 grs. of calomel every two hours, until a drachm of the former and half a drachm of the latter should be taken, if so much was requisite; and this to be given without regard to the pyrexial paroxysm.

It does not appear from the position taken in the chapter on leprosy that the author recognizes a primary nerve lesion in the abnormalities of the incipiency, by peripheral insensibility in spots.

The exhaustive dissertation on diphtheria takes in its scope all the phases of the subject ; yet we are impressed with the importance of emphasizing the early resort to a thorough mercurialization, as reported upon so favorably by several observers within the past few years; and likewise the inhalation of spirits of turpentine with tar, which are evidently attended with more salutary results than any other vapors or fumes which have been resorted to by practitioners in the grave disorder. We concur fully in the favorable estimate put upon chlorate of potash for the correction of the dyscrasia, and indorse the recommendation of the chloride of iron, especially in the latter stages of the disease. The *extreme* views of its contagiousness are not supported by the experience of others as to its extension, since the propagation may be accounted for generally, on the principle that like causes produce like effects in a given locality, and yet disinfection and seclusion are expedient.

In the well-digested views upon "Epidemic Cerebro-spinal Meningitis" we find nothing so definite in its controlling influence over the disease as has been claimed by other writers in behalf of the free use of the salicyliate of soda. Rabies and hydrophobia are brought forward as realities, the skepticism of Dulles to the contrary notwithstanding.

Anthrax is confounded with malignant pustule, though Littrè tells us "there is no real analogy between anthrax and malignant pustule or "charbon." In the *Dictionaire Therapeutique* of Bonchut and Despres this distinction is also clearly set forth.

In considering Beriberi the author ignores the treatise of Betholdi, in which this disease is referred to the nerve centres, connected with the great sympathetic system; yet the practical remedy, in a change of climate, is insisted upon in common with all who have been familiar with this malady in foreign lands.

Realizing the inadequacy of these passing reflections to convey a correct impression of the field embraced under the headings referred to, and the total failure to comply with the requirements of a criticism upon the undertaking as a whole, we have only to commend the various articles which remain unnoticed to the careful consideration of the reader. With the assurance that no individual effort could have accomplished so well what has been presented by the various authors, it is still a matter of regret that the experience and acumen of the editor should not have been more prominent in the moulding of the materials embraced in this volume; and it is to be hoped that his guardianship of the practical department in medicine may be more conspicuous in the forthcoming volumes of this work.

